# COVID-19 pandemic in sub-Saharan Africa: preparedness, response, and hidden potentials

**DOI:** 10.1186/s41182-020-00240-9

**Published:** 2020-06-17

**Authors:** Issideen Ayinla Osseni

**Affiliations:** 1 Department of Clinical Medicine, International School, Jinan University, 510632 Guangzhou, People’s Republic of China; 2 Department of Medicine, UAC Health Sciences Faculty, Cotonou, Benin

**Keywords:** COVID-19, Pandemic, Sub-Saharan Africa, Preparedness, Response, Potential

## Abstract

After the detection of coronavirus disease 2019 (COVID-19) first reported case in Nigeria, the virus has spread to all sub-Saharan Africa (SSA). Through different initiatives, SSA countries came together to create goal-driven taskforces to improve their responses against the virus. As COVID-19 raises major concerns over the scarcity of medical supplies, numbers of SSA countries adopted innovative solutions to fill in their shortage. This health crisis may come as an opportunity for SSA to demonstrate its pandemic readiness, responses, and reveal unknown potential.

To the editor,

On 11 March 2020, COVID-19 caused by SARS-CoV-2 was declared a pandemic by the World Health Organization (WHO) [1]. Nations across the globe respond swiftly and different mitigation measures were enforced such as travel and movement restriction, gathering prohibition, generalized or partial lockdown, handwashing, and good hygiene promotion. The first instance of COVID-19 in SSA was reported on 28 January 2020 in Nigeria [2], and it has now spread to all SSA. With a vulnerable health care system, SSA countries are faced with multiple health challenges arisen from the COVID-19 pandemic. For instance, to avoid escalation in the numbers of cases and having hospitals submerged with patients, SSA governments put in place mitigation measures that limit population movement, therefore, breaking the chain of contamination. As a result, patients suffering from endemic diseases such as HIV, tuberculosis (TB), and malaria have limited access to health services hence prone to rationalized their medicines. Consequently, this situation will contribute to the appearance of drug-resistant pathogens, increase complications, and mortality from these endemic diseases [3]. Another major concern was that the pandemic would prove difficult to be controlled in SSA because most countries have poor health systems and inadequate health infrastructures.

A report from the World Bank showed that in 2017, SSA spent 5.17% of its total GDP on health. This number is far less than the 9.89% spent by the Organization for Economic Co-operation and Development (OECD) countries [4]. Consequently, there is less money spent on the development of its healthcare system. This resulted in under-maintained infrastructure, shortage of health workers while those available are inadequately trained, scarce supplies of personal protective equipment, ventilators, intensive care units (ICU) beds, and other medical necessities [5].

## Preparedness and response

To avoid a potential health disaster, it was therefore important for SSA countries to take responsibility against the pandemic with the help of WHO, Non-Governmental Organizations (NGOs), and other healthcare run programs. Preventive measures such as the COVID-19 awareness campaign, debunking myths surrounding the disease, flight cancellations, gathering restrictions, handwashing, and good hygiene promotion were quickly enforced. Across SSA, governments have created presidential task forces for public health and pledged millions of dollars to be directed against the pandemic. On February 22, 2020, in response to the covenant reached by Africa Health Ministers during a meeting convened by the African Union Commission, the collaboration between Africa Centers for Disease Control and Prevention (Africa CDC) and the Southern Africa Center for Infectious Disease Surveillance (SACIDS) gave birth to Africa Taskforce on Coronavirus Preparedness and Response (AFTCOR) [6]. AFTCOR is a continent-wide strategy for COVID-19. It will build on existing monitoring systems and process the laboratory diagnosis and subtyping; surveillance, including screening at points of entry and cross-border activities; infection prevention and control in health care facilities; clinical treatment of people with severe COVID-19; risk communication; and supply chain management and stockpiles [7]. After the detection of their first few cases, some countries went on full lockdown while some decided to only contain affected areas. Meanwhile, many other SSA countries were having difficulties detecting COVID-19 cases because of inadequate testing capacities. On April 21, 2020, the Partnership to Accelerate COVID-19 Testing (PACT): Trace, Test & Track (CDC-T3) was launched by the African Union Commission and the Africa CDC [8]. This new initiative aims to strengthen the capacity to test for COVID-19 across Africa, with emphasis on countries that are in dire need. As a result, at least 10 million Africans, who would have not been tested otherwise, will get tested in the next 6 months.

## Detective work, adaptability, and innovative response

Even with the best mitigation measures in place, viruses are more difficult to deal with than other microorganisms, so early detection is the best way to curb COVID-19 exponential spread. After the detection of their first cases, Health officials across SSA began an extensive detective work looking for people that were on the same plane as the patients (COVID-19 carrier) and people that have been in contact with them. To alleviate this exhaustive labor, some SSA countries such as Nigeria have come up with innovative technology like Mobile Location Data tracking. This lessens health official’s efforts by either individualized or aggregated tracking the mobile phones of confirmed cases to trail the movement of suspected patients and their contacts [9]. Similar initiatives were launched in Benin, Ethiopia, and South Africa as part of the COVID-19 management strategy.

All over SSA, public-private partnerships are coming to life to channel scarce resources toward this pandemic. For instance, in Uganda, automotive manufacturer Kiira Motors Corporation has teamed up with the Makerere School of Public Health to develop inexpensive ventilators for critically ill coronavirus patients. These ventilators will still be valuable post-COVID-19 [10]. Likewise in Nigeria, the federal government is in talks for a partnership with Innoson motors to manufacture low-cost ventilators. Countries like South Africa and Ghana are also looking into producing cost-effective ventilators. Meanwhile, researchers from the Pasteur Institute in Senegal have developed a cheap and quick diagnostic test for the virus. It cost one US dollar and gives results in 10 min [11].

Despite economic meltdown, spiraling inflation, and political unrest over the past decade, universities in Zimbabwe are making medical supplies such as face masks, gloves, and hand sanitizers that meet standards amid generalized containment measures [12]. Whereas in Kenya, a country that barely produces any surgical mask before grab attention when a clothes factory shifted its assembly line towards manufacturing 30,000 surgical masks a day. This transformation came in line to fill the shortage of surgical masks after the government made them compulsory to be worn in public [13]. Also in Kenya, a 3D company is making 3D face shields and has also printed prototype for a ventilator adaptor that could allow doctors to treat either two or four patients at the same time.

To prevent COVID-19 spread, the government of South Africa has rolled out mobile testing units to avoid the movement of potentially infected people. These mobile laboratories are deployed nationwide for a large scale screening to trace and monitor the disease propagation [14]. Meanwhile, in Benin, the government took an unprecedented step toward E-learning to limit the movement of students in overcrowded public universities. The ministry of education created a platform of E-learning where students could interact with teachers on video, using internet cost-free, via local network [15].

Many more innovative and adaptive solutions came alive during this global crisis and to further support more flourishing efforts, Youth in Africa Initiative of the African Development Bank created the #AfricaVsVirus challenge. The goal of this approach was to develop tech and non-tech solutions to the most pressing challenges triggered by the coronavirus pandemic with the opportunity of winning up to 50,000 US dollars in in-kind prizes [16].

## Learning from experience

Endemic diseases such as malaria, tuberculosis, and HIV are well known in SSA. Over the past decades, SSA has been subjected to emerging viral diseases such as Ebola and Lassa fever. Although being a health burden, these emerging viral diseases have provided several SSA countries vital health lessons, knowledge, and expertise [17]. For instance, the establishment of Africa CDC which was modeled on the US Centers for Disease Control and Prevention was officially launched in January 2017 by the African Union after the 2014–2016 Ebola epidemic, to prevent and improve the surveillance and emergency response to infectious diseases [2]. During the past Ebola outbreak, National Infection Prevention Control (IPC) plans were developed and published in each country, with IPC task forces established to coordinate infection control efforts [18]. Learning from the past outbreak lead to the development and testing of evidence-based protocols to improve both patient management and the implementation of public health interventions. More importantly, SSA has learned the value of community engagement to help overcome cultural barriers to pass on educational messages for a successful implementation of all interventions.

## Conclusion

The coronavirus pandemic poses major challenges all over the world. To improve and strengthen their responses against COVID-19, SSA countries came together under the control of Africa CDC to create different taskforces. The shortage of medical supplies and personal protective equipment is created by the pandemic lead numbers of SSA countries to adopt innovative counter-measure. Countries that have never produce surgical masks, gloves, sanitizers, or ventilators before have turned to domestic production. However, an important need remains to ensure the long-term sustainability of all the efforts underway. SSA needs investments and political will to continue its upward path to align public health resources, public-private partnerships, and scientific expertise to prevent, control, and manage future outbreaks before they become an epidemic. Also, this is an opportune occasion for SSA to further research on the potential of Traditional Medicine.

## Data Availability

All data used are publicly available, and sources are cited throughout.

## Abbreviations

SSA: Sub-Saharan Africa
COVID-19: Coronavirus disease 2019
Africa CDC: Africa Centers for Disease Control and Prevention
WHO: World Health Organization
AFTCOR: Africa Taskforce on Coronavirus Preparedness and Response
PACT: Partnership to Accelerate COVID-19 Testing
SACIDS: Southern Africa Center for Infectious Disease Surveillance
OECD: Organization for Economic Co-operation and Development
IPC: National Infection Prevention Control
